# Percutaneous tibial nerve stimulation versus electrical stimulation with pelvic floor muscle training for overactive bladder syndrome in women: results of a randomized controlled study

**DOI:** 10.1590/S1677-5538.IBJU.2015.0719

**Published:** 2017

**Authors:** Carlo Vecchioli Scaldazza, Carolina Morosetti, Rosita Giampieretti, Rossana Lorenzetti, Marinella Baroni

**Affiliations:** 1 Operating Unit of Uro-gynecology;; 2 Clinical Phatology;; 3 Physical Medicine and Rehabilitation. ASUR, Area Vasta n 2, Jesi, Italy

**Keywords:** Transcutaneous Electric Nerve Stimulation, Pelvic Floor, Urinary Bladder, Overactive

## Abstract

**Introduction:**

This study compared percutaneous tibial nerve stimulation (PTNS) versus electrical stimulation with pelvic floor muscle training (ES + PFMT) in women with overactive bladder syndrome (OAB).

**Materials and Methods:**

60 women with OAB were enrolled. Patients were randomized into two groups. In group A, women underwent ES with PFMT, in group B women underwent PTNS.

**Results:**

A statistically significant reduction in the number of daily micturitions, episodes of nocturia and urge incontinence was found in the two groups but the difference was more substantial in women treated with PTNS; voided volume increased in both groups. Quality of life improved in both groups, whereas patient perception of urgency improved only in women treated with PTNS. Global impression of improvement revealed a greater satisfaction in patients treated with PTNS.

**Conclusion:**

This study demonstrates the effectiveness of PTNS and ES with PFMT in women with OAB, but greater improvements were found with PTNS.

## INTRODUCTION

Overactive bladder syndrome (OAB) is a chronic disease characterized by urinary urgency with or without urge incontinence, frequency and nocturia (1, 2). The prevalence of OAB in the general population is reported to be 14-16% (3, 4). The total cost for diagnosis and treatment of OAB in the USA, during 2000 was estimated to be about USD 12.6 billion, which is comparable to the cost of osteoporosis and gynecologic and breast cancers (4). Pelvic floor muscle training (PFMT) and electrical stimulation (ES) are some of the less invasive procedures for treating OAB symptoms. Therefore, they are used as first treatments (5-7). Percutaneous tibial nerve stimulation (PTNS) is a minimally invasive and effective therapy used both as first-line treatment, as well as in managing of unresponsive patients (8, 9). Nevertheless, in common practice, antimuscarinic agents are frequently used as first treatment although burdened by a low adherence and although these patients need protracted treatment with periodic controls. The aim of this study was to compare efficacy, safety, quality of life and patient’s satisfaction parameters in patients treated with two different therapies for OAB symptoms.

## MATERIALS AND METHODS

From September 2014 to May 2015, 60 consecutive women (mean age: 58.5 years, range 38-72) with OAB syndrome were enrolled in this randomized controlled study. All subjects previously underwent a detailed clinical evaluation, including a complete history and physical examination. Patients with stress incontinence, urinary tract infection, neurological disease, bladder lithiasis, genital prolapse higher than stage II on POP-Q system, pregnancy, diabetes mellitus, a history of anti-incontinence surgery and/or prolapse repair, pelvic tumors and previously treated with radiation therapy or antimuscarinic agents, and patients who were not cooperative, were excluded. The study was approved by the local ethics committee and all patients signed informed consent before starting treatment. Women were divided randomly into two groups of 30 patients each using online randomization (GraphPad QuickCalcs software: http://www.graphad.com/quickcalcs/randomize1) by an independent biostatistician who was unaware of treatments performed by patients and was not involved in the study. In group A, women underwent pelvic floor rehabilitation. The treatment consisted of ten sessions of electrical stimulation (ES) followed by pelvic floor muscle training (PFMT). The sessions were performed three times a week for one hour. ES was applied in short-term mode with vaginal probe for 30 minutes using biphasic square waves with 20Hz frequency for 30 sec., alternating at 5Hz also for 30 sec. Every seat was individually followed by a physiotherapist and patients were carefully instructed to perform a correct pelvic muscle contraction. After the ten sessions, the patients continued the exercises at home for six months. In group B, the women underwent PTNS twice a week for 30 min each for a total of 6 weeks. All patients were assessed one month after the end of the treatments performed at the physiotherapy clinics.

### Endpoints

Reduction in number of voids per 24 hours was considered the primary efficacy endpoint in this study. Secondary endpoints were: reduction in number of episodes of urge incontinence, reduction of episodes of nocturia, changes in patient perception of intensity of urgency, impact of OAB symptoms on patient’s quality of life and evaluation of improvement: number of voids per 24 hours, episodes of nocturia and urge incontinence were evaluated using a 3-day micturition diary. The Overactive Bladder questionnaire Short Form (OAB-q SF) was used to assess the impact of OAB symptoms on patient’s quality of life. The questionnaire consists of 6 items related to symptoms with 6 possible options ranging from “not at all” (score 1) to “a very great deal” (score 6), and a health-related quality of life scale with 13 items, with 6 response options ranging from “none of the time” (score 1) to “all of the time” (score 6). Urgency was assessed by the Patient Perception of Intensity of Urgency Scale (PPIU-S) consisting of a 5-point scale from 0 (no urgency) to 4 (urge incontinence). Improvement was evaluated with the Patient Global Impression of Improvement questionnaire (PGI-I). The PGI-I is a validated generic tool for assessment of the overall improvement or deterioration that patients experience following the treatment. It is a 7-point scale from “very much improved (score 1), to “very much worse” (score 7). The micturition diary, OAB-q SF and PPIU-S, were completed before and after treatment. PGI-I was performed only at the end of treatment.

#### Statistical analysis

Statistical analysis was performed using the MedCalc software package (version 14.12.0). Data are expressed as means±SD. Comparisons were carried out using the Wilcoxon test for paired samples, the Mann-Whitney for independent samples and the Chi square test (X2 test). A p value of <0.05 was considered significant. Data were assessed by a researcher not involved in the study protocol.

## RESULTS

All 60 patients enrolled in the two groups were evaluable at the end of the study. No significant side effects were found with the two treatments. A reduction in the number of daily micturitions was found both with ES+PFMT and with PTNS with a significant difference in the group of patients undergoing PTNS (Table-1). Anyway, the number of daily micturitions did not show significant differences when comparing the results obtained after therapy in the two groups of patients (Table-1). Nocturia and urge incontinence showed improvements in both groups with significant differences only in patients treated with PTNS. Also, when the two groups were compared after treatment, women treated with PTNS showed statistically significant improvement compared to those treated with ES+PFMT (Table-1). Voided volume significantly improved in the two groups of patients with more evident results in the group treated with PTNS, also in the post treatment comparison. The quality of life of patients evaluated with OAB-q SF showed significant improvements in groups, both with ES+PFMT and with PTNS (Table-2). When comparing post treatment data, patients treated with PTNS showed better results than those treated with ES+PFMT. Improvements were found in PPIU-S in the two groups of patients with a statistically significant difference in women undergoing PTNS when the two groups were compared following treatment (Table-2). The PGI-I showed improvements in both groups of patients, with a significant difference in women treated with PTNS (Table-2).

**Table 1:** 3-day micturition diary.

		Group A FISIO					Group B SANS					
Patients, n		30					30					
Mean age		57.31					59.69					
Median (range)		60 (38-69)					62 (40-72)					
Daily micturition		**Before**		**After**			**Before**			**After**		
Mean±SD		11.15±2.07		9.03±1.68			11.25±1.13			9.00±2.02		
Median (range)		9 (7-18)		9 (6-12)			11 (9-14)			9 (6-13)		
p		0.0620					0.0307					
After Group A vs. After Group B: p=0.3758												
Nocturia			**Before**			**After**			**Before**	**After**		
Mean±SD			2.62±1.00			1.54±0.93			2.50±1.02	1.45±1.02		
Median (range)			3 (1-4)			2 (0-3)			3 (0-4)	2 (0-3)		
p			0.1683						0.0201			
After Group A vs. After Group B: p=0.049												
**Urge incontinence**	**Before**				**After**			**Before**			**After**	
Mean±SD	2.54±0.63				2.00±0.68			3.05±0.97		1.45±1.00		
Median (range)	3 (1-3)				2 (1-3)			3 (0-4)		2 (0-3)		
p	0.1293							0.0009				
After Group A vs. After Group B: p=0.0251												
Voided Volume (*)			**Before**			**After**		**Before**				**After**
Mean±SD			136.75±11.92			157.92±10.30		140.21±13.50				171.42±12.68
Median (range)			133.5(115-160)			155 (140-180)		135 (120-165)				175 (145-190)
p			0.0048					0.0003				
After Group A vs. After Group B(**): p=0.0222												

(*) Wilcoxon test for paired samples

(**) Mann-Whitney for independent samples

**Table 2:** Overactive Bladder questionnaire Short Form (OAB-qSF), Patient Perception of Intensity of Urgency Scale (PPIUS) and Patient Global Impression of Improvement questionnaire (PGI-I).

			Group A			Group B				
Patients, n			30			30				
OAB-qSF (6 items)(*)			**Before**		**After**	**Before**			**After**	
Mean±SD			19.46±3.13		15.77±5.48	21.35±2.57			12.90±2.93	
Median (range)			20 (14-24)		16 (8-28)	22 (14-25)			13.5 (6-18)	
p			0.0420			<0.0001				
After Group A vs. After Group B (**): p=0.0172										
OAB-qSF (13 items) (*)		**Before**			**After**	**Before**			**After**	
Mean±SD		36.85±13.02			29.38±9.32	44.40±8.51			24.85±5.96	
Median (range)		39 (15-54)			29 (14-50)	45.5 (22-64)			24 (12-39)	
p		0.0420				<0.0001				
After Group A vs. After Group B (**): p=0.0295										
**PPIUS**		**Before**			**After**		**Before**			**After**
Mean±SD		2.77±0.80			2.00±0.68		3.00±0.63			1.75±0.77
Median (range)		3 (2-4)			2 (1-3)		3 (1-4)			2 (1-3)
p		0.1014					0.0001			
After Group A vs. After Group B: p=0.0459										
**PGI-I**	**Before**			**After**				**Before**	**After**	
Mean±SD	2.85±0.36							2.30±0.78		
Median (range)	3 (2-3)							2 (1-4)		
p	0.0415									

(*) Wilcoxon test for paired samples

(**) Mann-Whitney for independent samples

## DISCUSSION

Less invasive procedures represent the first-line treatment recommended by the Consensus Conference on Urinary Incontinence in Adults and by the guidelines of the American Urological Association/Society of Urodynamics, Female Pelvic Medicine and Urogenital Reconstruction (AUA/SUFU) for therapy of OAB (5, 6). However, in common practice, antimuscarinic agents are frequently the first treatment considered for treating OAB (6), but results have shown that a high percentage of patients treated with these drugs discontinued therapy (10-13).

Pelvic floor muscle training (PFMT) was popularized by Arnold Kegel in 1946 for the management of urinary incontinence (14), and then it was also used in the treatment of urge incontinence and overactive bladder (15). Pelvic floor muscle contraction can be used to occlude the urethra and prevent urine loss during detrusor contraction but it can improve bladder control by inhibiting or suppressing bladder contraction which also changes the patient’s behavior (16). PFMT was used alone or in combination with behavioral and cognitive therapy (6), vaginal cones, bladder training, ES, drug treatment, continence pessary, heat and steam generating sheet (HSGS) (17) and assisted with EMG-biofeedback (18). PFMT has shown good results in reducing urge incontinence, urinary frequency and nocturia (15, 18-21). ES was particularly effective in the treatment of OAB symptoms (20) and is considered by some authors to be more effective than drug treatment, permitting an effective reduction or inhibition of detrusor activity by stimulating afferents of the pudendal nerve (22). PTNS is a form of “neuromodulation” (23); it is a safe, minimally invasive and effective treatment for managing refractive OAB (9, 23). Randomized studies comparing PTNS versus anticholinergic agents have shown that the efficacy of PTNS is comparable or superior to anticholinergic agents in controlling OAB symptoms but with a better side effect profile (8, 24-28). To our knowledge, studies carried out comparing percutaneous tibial nerve stimulation with pelvic floor muscle training and electrical stimulation are not described in the literature. The results of this study relate to the short term efficacy of these two different treatments. The data obtained, even though carried out on a moderate number of patients, highlight a complete adherence by all the women to the performed treatments. This is a result that should be emphasized as it is a chronic symptom for which great adherence to therapy is required by patients. These results are due mainly to the total absence of side effects. Furthermore, the low cost to patients of the therapy and the constant and reassuring presence, during treatments, of a physiotherapist or medical specialist, represent important factors in patients who often have psychological problems secondary to OAB. Improvements were found in both groups in all symptoms evaluated with 3-day micturition diary, but PTNS showed greater effectiveness than ES+PFMT. When evaluating results obtained following therapy in the two groups of patients, PTNS showed statistical significant improvements compared to women treated with ES+PFMT in all parameters except daily micturition, which in any case registered greater results in patients treated with PTNS. In the same way, the data obtained by comparing completed questionnaires also confirmed better results with statistically significant improvements in patients treated with PTNS.

## CONCLUSIONS

ES and PFMT represent some of the less invasive but effective procedures recommended for OAB treatment. In common practice, PTNS is a procedure used especially when other treatments have failed. For its effectiveness and its minimal invasiveness PTNS should be considered as a first-line treatment.
